# Evaluating the correlation between pediatric exposure rates and common body size surrogates in fluoroscopy

**DOI:** 10.1002/acm2.70659

**Published:** 2026-07-07

**Authors:** Keith J. Strauss, Elanchezhian Somasundaram, Joseph G. Meier, Samuel L. Brady

**Affiliations:** ^1^ Department of Radiology Cincinnati Children's Hospital Medical Center Cincinnati Ohio USA; ^2^ Department of Radiology University of Cincinnati School of Medicine Cincinnati Ohio USA

**Keywords:** fluoroscopy, patient size metrics, pediatric, quality assurance, radiation dose, reference air kerma

## Abstract

**Purpose:**

To quantify the degree of correlation between pediatric radiation exposure rates and commonly used body size surrogates: anteroposterior (AP) or lateral (LAT) body thickness, weight, height, body mass index (BMI), body surface area (BSA), and age, for general fluoroscopy (GF) and cardiac fluoroscopically guided interventional (cardiac FGI) examinations, as a way to identify the most predictive surrogate to optimize dose management and exposure control across a wide range of pediatric body sizes.

**Methods:**

This retrospective study included 6447 GF examinations, from 4452 pediatric patients, and 2968 cardiac FGI examinations, from 1471 patients, aged from birth to 21 years. Reference air kerma (RAK) data, collected from a radiation dose index monitoring database, were corrected using calibration measurements with accuracy better than ± 5%. Exposure rate was calculated as RAK divided by total fluoroscopy time. Patient body size surrogates: measured AP or LAT thickness, age, weight, height, BMI, and BSA were extracted or computed from recorded data. Correlations between log‐transformed exposure rates and body size surrogates were evaluated using Pearson's correlation coefficients. Steiger's *Z*‐test was applied to assess statistically significant differences between dependent correlations.

**Results:**

For GF examinations, AP thickness correlated most strongly with exposure rate (*r* = 0.691), followed by BSA (*r* = 0.574) and weight (*r* = 0.568). BMI showed the weakest correlation (*r* = 0.446). For cardiac FGI examinations, weight demonstrated the highest correlation with exposure rate in both frontal (*r* = 0.689) and LAT (*r* = 0.794) planes, with BSA and LAT thickness performing similarly. All other correlations were significantly lower (*p* < 0.001). The superior performance of AP thickness in GF reflects its direct relationship to X‐ray attenuation, whereas variable geometry and positioning during cardiac FGI favor weight as a more stable predictor.

**Conclusions:**

For pediatric fluoroscopy, AP thickness best predicts exposure rate in GF, while weight is the most reliable surrogate for cardiac FGI procedures. Selecting modality‐specific size metrics enhances pediatric dose optimization and supports consistent radiation exposure management across all patient sizes.

## 1 INTRODUCTION

Examinations of pediatric patients using imaging modalities such as general fluoroscopy (GF) and cardiac fluoroscopically guided interventional (cardiac FGI) procedures are performed daily to support appropriate medical care. In these procedures, an X‐ray beam passes through the patient, and as it traverses tissue, photons of varying energy are absorbed by a process known as attenuation. Properly accounting for attenuation is essential because it affects both the radiation dose (i.e., patient radiation risk) and the quality of the resulting image needed for accurate diagnosis. In pediatric imaging, this balance between image quality and radiation dose is particularly critical due to the wide variation in patient size and sensitivity to radiation.^1^, ^2^

Image quality depends on maintaining a minimum number of photons reaching the detector; below this threshold, the image becomes diagnostically unacceptable. The total X‐ray energy that must be delivered to the detector depends on how much is attenuated by the patient, which in turn is determined primarily by patient thickness. In pediatric imaging, anteroposterior (AP) trunk thickness can range from as little as 5 cm in neonates to more than 42 cm in older adolescents.^3^ This wide variation in body size requires corresponding adjustments in X‐ray output to achieve acceptable image quality while minimizing dose.^4^, ^5^, ^6^

Careful management of X‐ray attenuation across the wide range of pediatric body sizes is therefore essential. Yet it remains unclear which body size surrogate best correlates with the radiation output reported by the imaging device for GF and cardiac FGI examinations. A review of the literature found the use of the following body size surrogates: physical thickness,^4^, ^5^, ^7^ weight,^8^, ^9^, ^10^, ^11^, ^12^ height,^11^, ^12^ body mass index (BMI),^11^, ^12^, ^13^, ^14^ body surface area (BSA),^11^, ^12^ or age^15^, ^16^; no published data directly compared the strength of these correlations in pediatric populations for GF and cardiac FGI imaging procedures.

Intuitively, body thickness should strongly correlate with machine radiation output, as it most directly reflects the physical parameter of attenuation; however, measuring body thickness is perceived to be cumbersome in clinical practice,^17^ prompting interest in simpler, faster surrogate measures of patient size. Consequently, in the absence of a more accurate or practical alternative, many imaging centers continue to rely on patient weight to select appropriate imaging protocols.^18^

Because X‐ray attenuation depends primarily on path length through tissue, we hypothesize that patient body thickness will show the strongest correlation with radiation output in pediatric GF and cardiac FGI procedures, compared with other size surrogates such as weight, height, BMI, BSA, or age. This study tests that hypothesis by quantifying the degree of correlation between radiation output rate and common body size surrogates. The overarching goal is to support improved dose optimization and image quality for patients from birth through age 21. Enhanced pediatric patient care is achieved by configuring the fluoroscope so that technique factors and other control parameters^1^ adjust appropriately as a function of radiation output rate. This reconfiguration requires a collaboration between the fluoroscope's manufacturer, medical physicist, and clinical end users prior to first clinical use.^19^ This process is best supported when clinical end users and the medical physicist rely on a size surrogate that closely correlates with the radiation output rates used for pediatric patients of varying sizes.

## 2 METHODS

### 2.1 Data collection

This retrospective study anonymized all patient data and was compliant with the Health Insurance Portability and Accountability Act. The Institutional Review Board waived the need for consent for this retrospective study. No AI generative models were used in this work.

Two distinct datasets were analyzed. The first set collected the Reference air Kinetic Energy Released in Mass (KERMA) without backscatter,^20^ that is, *K_a,r_*, hereafter referred to as RAK for 4452 pediatric patients across 6447 GF examinations. Each examination was categorized as a Gastrointestinal (GI), Voiding Cystourethrogram (VCUG), or Tube Placement (TP) study involving the trunk.^4^ For each patient, the AP and/or lateral (LAT) body thickness was measured to the nearest whole centimeter using mechanical calipers, providing an estimate of the X‐ray path length through the body.

The second dataset comprised 1471 pediatric patients who collectively underwent 2968 cardiac FGI examinations. These examinations represented a broad spectrum of diagnostic and interventional studies performed in the cardiac catheterization laboratory; a detailed list of procedure types is available in Table 3 of a previous publication.^5^ RAK values were recorded from both the frontal and LAT imaging planes: frontal plane RAK was available for all 2968 examinations, while LAT plane RAK was available for 2849 examinations. Because manual AP thickness measurements were difficult when the patient table was not flat, only the LAT thoracic thickness at the level of the heart was measured. AP chest thickness values at the cardiac level published from clinical CT images for patients ranging from newborns to 21 years were matched to the measured LAT thicknesses in this study.^3^

During data collection for both datasets, pediatric patient thickness (cm), age (yr), weight (kg), and height (m) were collected, which also allowed the calculation of BMI = mass/height^2^ (kg/m^2^),^21^ and BSA = [height(cm) * mass(kg)/3600]^0.5^ (m^2^).^22^ The RAK and various pediatric patient size surrogates for both datasets were stored in a Radiation Dose Index Monitoring (RDIM) database (Clinical Microsystems Corporation, Riva, MD, USA). The first and second sets were collected during examinations performed between October 1, 2016, through December 31, 2019, and January 1, 2016, through March 31, 2022, respectively. All examinations with complete data in the database, within the period of the study, were included. During the six years of data collection, the five general fluoroscopes and three biplane fluoroscopic guided cardiac interventional units, see Table 1, used in this study were not replaced nor was their configuration changed.

**TABLE 1:** Basic specifications and quantity of each imaging device.

Type	*N*	Manufacturer	Model	Reference point	
GF^2^	5	Philips Healthcare Solutions Amsterdam, Netherlands	Easy diagnostic, under‐table X‐ray tube, tilt table	65 cm	
				Isocenter	Int Ref Pt
FGI^3^	2	Philips Healthcare Solutions Amsterdam, Netherlands	Allura Xper FD10 with clarity image processing: Biplane unit	Frontal: 76.5 cm Lateral: 76.6 cm	Frontal: 61.5 cm Lateral: 61.6 cm
FGI^3^	1	Philips Healthcare Solutions Amsterdam, Netherlands	Allura Xper FD20 with clarity image processing: Biplane unit	Frontal: 81 cm Lateral: 76.6 cm	Frontal: 66 cm Lateral: 61.6 cm

Abbreviations: FGI, fluoroscopic guided intervention in Cardiac Catheterization Lab; GF, general fluoroscopic examination in Radiology; Int Ref Pt, distance from focal spot to a point 15 cm back from the isocenter toward the X‐ray tube^24^; Isocenter, Distance from focal spot to gantry's point of rotation; *N*, number of imaging units, Reference point, Distance from focal spot to entrance plane of patient.

### 2.2 Patient exposure rates

RAK was chosen over the KERMA Area Product (KAP) as the radiation output index to avoid introducing a second variable in the analysis, namely area of the X‐ray field.

For the first dataset, patients were positioned supine on a tilt table unit, maintaining a fixed distance from the X‐ray tube focal spot and thereby eliminating the need for inverse‐square law corrections to RAK. Calibration correction factors^23^ appropriate to the year of the clinical study were derived from radiation measurements obtained during annual compliance testing of each of the five fluoroscopes listed in Table 1 and applied to the cumulative RAK values stored in the RDIM database. These corrections reduced the uncertainty in reported RAK values to within ± 5%. Finally, the ratio of the corrected total RAK to the total fluoroscopy time for each examination was calculated to yield a radiation output rate, expressed in µGy/min, hereafter referred to as patient exposure rate.

For the second dataset, calibration correction factors,^23^ determined as described in the previous paragraph, were applied to the cumulative displayed RAK values for each of the six planes listed in Table 1, reducing the measurement error to within ± 5%. This corrected RAK was at the interventional reference point (IRP), which places the entrance plane of an average‐sized adult at 15 cm from the isocenter (ISO) toward the focal spot.^24^ Since a pediatric patient's entrance skin plane is typically farther from the focal spot than the adult defined IRP, the displayed RAK stored in the RDIM database was corrected by applying the inverse square law using Equation 1:^5^

(1) $$ISC={\left[\frac{IRP}{ISO-\frac{\left(AP+LAT\right)}{4}}\right]}^{2}$$

where *ISC* denotes the inverse‐square law correction factor, *IRP* (Table 1) represents the interventional reference point, and *ISO* (Table 1) denotes the isocenter of the fluoroscope gantry. *AP* and *LAT* correspond to the AP and LAT thoracic thicknesses, respectively, measured in centimeters. The term *(AP + LAT)/4* estimates the average patient radius during the examination, enabling determination of the RAK at the entrance skin plane for the pediatric patients included in this study. The inverse‐square law correction assumes that the center of the heart is positioned at ISO.^5^ The ratio of the corrected total RAK to the total fluoroscopy time of the examination was calculated to create a radiation output rate expressed in mGy/min, hereafter referred to as patient exposure rate.

For both datasets, pediatric GF and cardiac FGI examinations, the ratio of total RAK during fluoroscopy and acquisitions to fluoroscopy time represents a reasonable index of exposure rate, under the assumption that the number of radiographic acquisitions increases proportionally with fluoroscopy time.

### 2.3 Patient exposure rate versus body size surrogates

Based on the log‐transformed simple linear fit equation developed for GF exposure rate as a function of patient size,^4^ the equation for calculating RAK/time versus different body size surrogates is given by Equation 2:^4^

(2) $$\mathrm{Exposure}\;\mathrm{Rate}\left(x\right)=\exp\left(m*x+c\right)$$

where *Exposure Rate* denotes the patient exposure rate (i.e., RAK / fluoroscopy time), *c* denotes the intercept value of the linear exposure rate fit, *m* denotes the slope of the fit, and *x* denotes the body size index, for example, thickness, age, height, weight, etc.

### 2.4 Statistical analysis

Patient exposure rates for GF and cardiac FGI examinations were log‐transformed to linearize the relationship between exposure rates and body size surrogates, as radiation output rates often exhibit an exponential relationship with patient size. Pearson's correlation coefficients (*r*) were then calculated between each patient variable and the log‐transformed exposure rate parameter for the two datasets. Finally, the correlation between the log‐transformed patient exposure rates and a primary patient size surrogate, AP thickness for GF, LAT thickness for cardiac c was compared against correlations with other patient size surrogates using Steiger's *Z*‐test^25^ for dependent correlations from the cocor^26^ package in R.^27^

## 3 RESULTS

**FIGURE 1:**
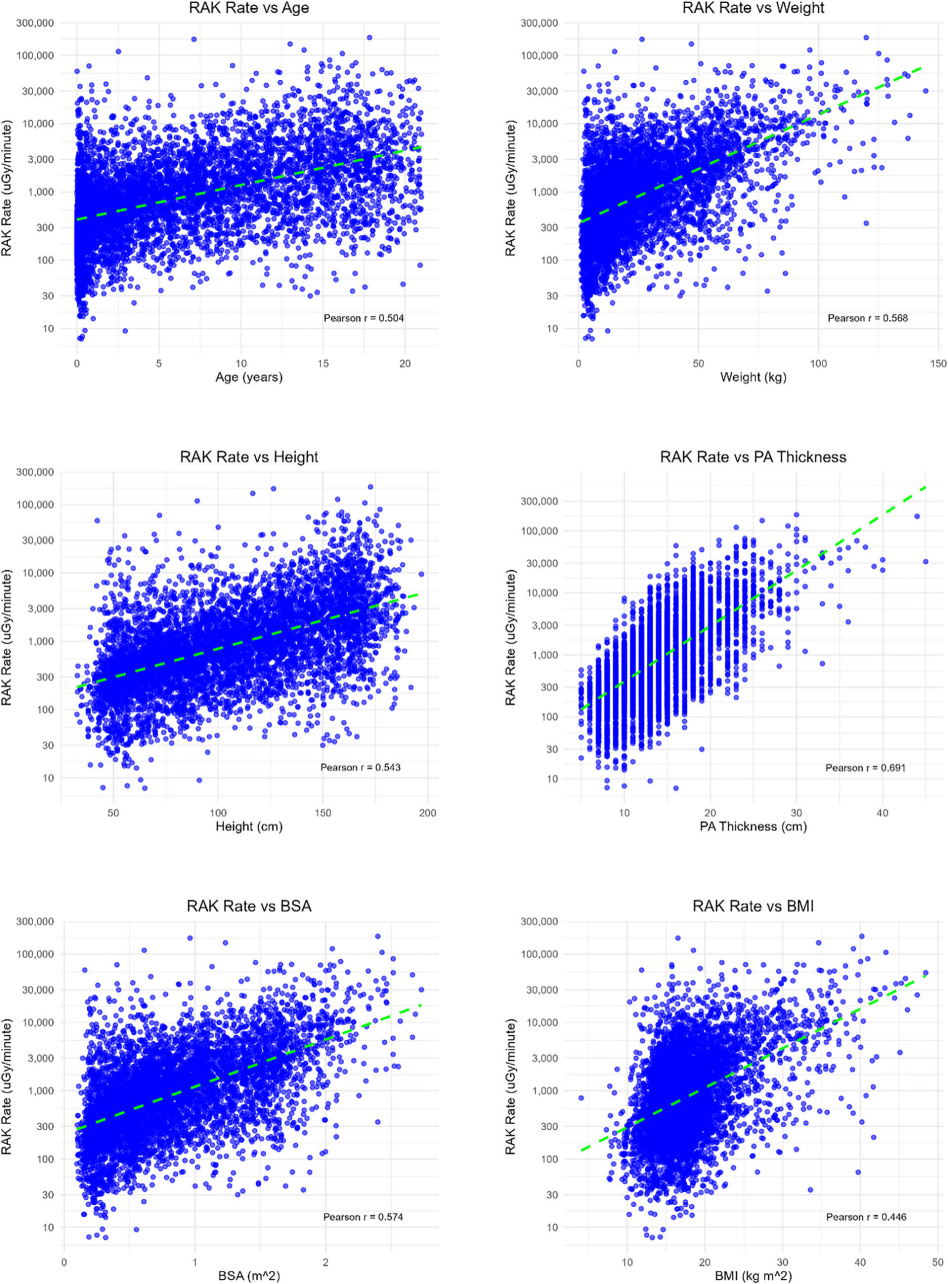
Correlation of pediatric reference air KERMA (RAK) rates during GF with body size surrogates. Scatterplots show the logarithmic RAK rate versus age, height, weight, measured AP thickness, BMI, and BSA for patients undergoing GF examinations. Blue points represent individual patient examinations; the green dashed line indicates the simple linear regression fit.

The strength of association between patient exposure rate and various body size surrogates was evaluated using Pearson's *r* statistic, as summarized in Tables 2, 3, 4. Figure 1 presents linear regression plots of the log‐transformed exposure rates for GF examinations versus age, weight, height, AP thickness, BSA, and BMI. The green dashed line in each plot represents the simple linear regression fit. As shown in Figure 1, the five body size surrogates other than BMI track along the simple linear regression fit, which suggest a stronger correlation with patient exposure rate than BMI.

**TABLE 2:** Degree of correlation: GF.

Number of examinations = 6447		
Metric	Pearson's *r*	*p*‐value (Comparison to Size Metric)
**Size PA (cm)**	0.691	–
**BSA (m^2)**	0.574	< 0.00001
**Weight (kg)**	0.568	< 0.00001
**Height (cm)**	0.543	< 0.00001
**Age (Years)**	0.504	< 0.00001
**BMI (Kg/m^2)**	0.446	< 0.00001

**TABLE 3:** Degree of correlation: Cardiac fluoroscopy guided interventional (FGI) frontal plane.

Number of examinations = 2968		
Metric	Pearson's *r*	*p*‐value (Comparison to Size Metric)
**Weight (kg)**	0.689	<0.00001
**BSA (m^2)**	0.677	<0.00001
**Size LAT (cm)**	0.657	–
**Age (Years)**	0.639	0.0037
**Height (cm)**	0.610	<0.00001
**BMI (Kg/m^2)**	0.547	<0.00001

**TABLE 4:** Degree of correlation: Cardiac fluoroscopy guided interventional (FGI) LAT plane.

Number of examinations = 2849		
Metric	Pearson's *r*	*p*‐value (Comparison to Size Metric)
**Weight (kg)**	0.794	0.016
**BSA (m^2)**	0.792	0.021
**Size LAT (cm)**	0.784	–
**Age (Years)**	0.754	<0.00001
**Height (cm)**	0.736	<0.00001
**BMI (Kg/m^2)**	0.638	<0.00001

In Tables 2, 3, 4, patient body size surrogates are arranged in descending order of correlation strength with the patient exposure rate. The *p*‐value in each row tests whether the correlation between the listed surrogate and the exposure rate differs significantly from that obtained using patient thickness. Across all tables, the difference in correlation between each body size surrogate and patient thickness is statistically significant. Among the evaluated parameters, AP thickness showed the strongest correlation with patient exposure rate for GF examinations (*r* = 0.691), whereas patient weight demonstrated the strongest correlation for cardiac FGI examinations in both frontal (*r* = 0.689) and LAT (*r* = 0.794) planes.

## 4 DISCUSSION

This study examined the relationship between pediatric radiation exposure rates and several body size surrogates: patient thickness, weight, height, age, BMI, and BSA for GF and cardiac FGI examinations. The analysis demonstrated that patient body thickness exhibited the strongest correlation (*r* = 0.691) with patient exposure rate for GF procedures. These findings align with the physical principles of X‐ray attenuation: as patient thickness increases, a higher X‐ray output is required to maintain adequate image quality, leading to a corresponding increase in measured patient exposure rates. Although body thickness most directly reflects X‐ray path length, its measurement can be cumbersome in daily clinical practice.^17^ Consequently, many imaging centers rely on surrogate size measures, such as patient weight, to select exposure parameters. In this study, weight showed a slightly lower but still strong correlation with patient exposure rate (*r* = 0.568), suggesting it may serve as a practical alternative when direct thickness measurements are unavailable during GF procedures.

Among the remaining indices, BSA showed the second‐highest correlation after thickness (*r *= 0.574), but its calculation requires both weight and height, making it less convenient for routine clinical use. Age and BMI, in contrast, showed poor correlation with patient exposure rates. This finding highlights the wide variability in body habitus among pediatric patients of similar ages. For example, AP trunk thicknesses ranging from 6–18 cm in 2‐year‐olds and 13–25 cm in 15‐year‐olds^3^ demonstrates the limited predictive value of age‐based protocols.

When considering pediatric cardiac FGI, results from this study demonstrate that exposure rate is most strongly correlated with patient weight, as indicated by the Pearson's *r* values reported in Tables 3 and 4. The next best correlation was observed with BSA; however, BSA showed a correlation comparable to that of patient thickness and weight (Table 4). Because BSA requires accurate measurements of both height and weight at the time of the procedure to be calculated, patient weight remains the preferred body size index. When available, patient thickness is also an appropriate metric for correlating exposure rate in either the frontal or LAT imaging planes. Age, height, and BMI demonstrated weaker correlations for cardiac FGI examinations, consistent with findings from GF examinations.

The lack of superior statistical performance for patient thickness in cardiac FGI likely reflects several procedural and geometric factors. First, although operators ideally adjust table height to position the heart at isocenter, this may not have occurred consistently across all cases included in the dataset. Second, the use of various compound angulations of the frontal and LAT imaging planes introduces substantial variation in the source‐to‐skin distance during the procedure, which was not accounted for in the inverse square correction. In this study, a single average skin distance was assumed for inverse square law corrections of exposure rate. Consequently, the average exposure rate estimates are less precise for cardiac FGI cases than for GF examinations. Under these conditions, patient weight serves as the most reliable surrogate size metric for correlation with exposure rate.

The above correlations require that the body size surrogate used is determined near to the time of the examination, especially for growing children. Measured patient thickness at the time of the examination are ideal. Body size surrogate's dependent on weight or height of the patient, which are typically provided to the RDIM database from other hospital databases may not be current relative to the date of the examination

Mann et al.^28^ identified several benchmarking challenges associated with using RAK values reported in Radiation Dose Structured Reports (RDSRs), including inconsistent reference‑point definitions and substantial variability in RAK accuracy across manufacturers and system models. To avoid these limitations, our study did not rely on uncorrected RDSR‑reported RAK values. Instead, we evaluated RAK at the patient's skin‑entrance plane by applying annually measured calibration correction factors obtained at a defined, standardized location^22^. These annual calibration measurements ensured that displayed RAK values were accurate to within ± 5%, a level of accuracy not achieved by approximately 50% of the RDSR‑reported RAK values in the Mann study.^28^

Other sources of RDSR inaccuracy highlighted by Mann et al.^28^ include heterogeneity among manufacturers and wide variation in system age. These factors were mitigated in our study because all fluoroscopes were manufactured by a single vendor and were less than seven years old at the conclusion of the study period. Mann et al.^28^ also identified the failure to capture elevated levels of radiation exposure from non‐fluoroscopic X‐ray tubes as a potential source of error. This error did not apply in our study, as all radiation exposure during fluoroscopy and during acquisitions with elevated exposure rates during both GF and cardiac FGI pediatric procedures were produced exclusively by the fluoroscopic X‐ray tubes and captured in the displayed KAP and RAK.

The degree of correlation between body size surrogates and patient exposure rate depends in part on the consistency with which protocol settings are applied by clinical staff. In our study, all personnel used specific, carefully designed protocols that adjust the fluoroscope's radiation output rate for GF and cardiac FGI procedures as a function of defined pediatric body thicknesses.^3^ As a result, for a given patient size, the exposure rate was generally independent of the operator. Overall, the consistent use of these well‑designed protocols likely strengthened the observed correlations between patient exposure rates and the selected body size surrogates.

A review of the literature revealed a paucity of data directly comparing the strength of correlations among body size surrogates in pediatric populations for fluoroscopic modalities. In adult populations undergoing cardiac FGI, two studies reported findings like those observed in our work. In these studies, the authors compared body size surrogates to dose‐area product (DAP). Koh et al.^12^ reported correlations between DAP and BSA (*r* = 0.222) and weight (*r* = 0.210). Similarly, Manicardi et al.^11^ reported that weight (*r* = 0.342) and BSA (*r* = 0.304) were the strongest correlates of radiation output in their adult cohort.

This study has limitations. Cumulative RAK was divided by fluoroscopy time for both GF and cardiac FGI examinations to obtain an exposure rate, which does not factor in elapsed time during radiographic acquisitions. However, it is not unreasonable to expect that the total contribution to patient exposure from radiographic acquisitions should increase proportionally with increases of fluoroscopy time during the examination. The data analysis of this study did not address the individual compound angles of the fluoroscope's imaging planes for the thousands of examinations in the study. Rather, a single average distance for each study of the entrance plane of the patient from the focal spot was estimated assuming the measured thickness of the patient and the heart being centered on the isocenter of the imaging planes. All the data in this study is from a single academic tertiary care medical center, but this study is focused on the correlation of patient exposure rates to body size surrogates, as opposed to actual patient exposures. While other patient dose indices, especially peak skin dose, are more directly related to patient risk than the ratio of RAK to total fluoroscopy time, the use of patient exposure rate is a starting point from which a qualified medical physicist can calculate the desired patient dose estimate.^29^

## 5 CONCLUSION

This study identified the strongest correlations between six different body size surrogates and pediatric patient exposure rates for GF and cardiac FGI examinations. For GF examinations, the AP patient thickness demonstrated the highest correlation with exposure rate, whereas for cardiac FGI examinations, patient weight showed the strongest relationship. The secondary correlation surrogates were either BSA or patient weight for GF examinations while either BSA or patient thickness for cardiac FGI examinations. Selecting the most appropriate body size surrogate to correlate with pediatric exposure rate represents an important step toward improving dose management and image quality optimization for pediatric patients ranging from newborns to 21 years of age.

## AUTHOR CONTRIBUTIONS

All four authors made substantial contributions to the conception and design of the manuscript; actively participated in the acquisition, analysis, and interpretation of the data; drafted and/or critically revised the manuscript for intellectual content; approved the final submitted version; and agree to be accountable for all aspects of the work.

## CONFLICT OF INTEREST STATEMENT

The authors declare no conflicts of interest

## FUNDING INFORMATION

No funding supported this project.
